# Analysis of mRNA-miRNA interaction network reveals the role of CAFs-derived exosomes in the immune regulation of oral squamous cell carcinoma

**DOI:** 10.1186/s12885-023-11028-5

**Published:** 2023-06-26

**Authors:** Wei-Zhou Wang, Xue Cao, Li Bian, Yue Gao, Ming Yu, Yi-Ting Li, Jian-Guo Xu, Yang-Hao Wang, He-Feng Yang, Ding-Yun You, Yong-Wen He

**Affiliations:** 1 Yunnan Key Laboratory of Stomatology, Kunming Medical University, Kunming, Yunnan China; 2 Department of Orthopedics, The First Affiliated Hospital of Kunming Medical University, Kunming, Yunnan China; 3 Department of Laboratory Animal Science, Kunming Medical University, Kunming, Yunnan China; 4 Department of Pathology, The First Affiliated Hospital of Kunming Medical University, Kunming, Yunnan China; 5 College of Bioinformatics Science and Technology, Harbin Medical University, Harbin, Heilongjiang China; 6 Yunnan Key Laboratory of Stem Cell and Regenerative Medicine, Biomedical Engineering Research Center, Kunming Medical University, Kunming, Yunnan China; 7 Department of Dental Research, The Affiliated Stomatological Hospital of Kunming Medical University, Kunming, Yunnan China; 8 Qujing Medical College, Qujing, Yunnan China

**Keywords:** Cancer-associated fibroblasts, Exosomes, Oral squamous cell carcinoma, mRNA-miRNA interaction network analysis, Immunomodulation

## Abstract

**Background:**

Cancer-associated fibroblasts (CAFs) have significant tumor regulatory functions, and CAFs-derived exosomes (CAFs-Exo) released from CAFs play an important role in the progression of oral squamous cell carcinoma (OSCC). However, a lack of comprehensive molecular biological analysis leaves the regulatory mechanisms of CAFs-Exo in OSCC unclear.

**Methods:**

We used platelet derived growth factor-BB (PDGF-BB) to induce the transformation of human oral mucosa fibroblast (hOMF) into CAFs, and extracted exosomes from the supernatant of CAFs and hOMF. We validated the effect of CAFs-Exo on tumor progression by exosomes co-culture with Cal-27 and tumor-forming in nude mice. The cellular and exosomal transcriptomes were sequenced, and immune regulatory genes were screened and validated using mRNA-miRNA interaction network analysis in combination with publicly available databases.

**Results:**

The results showed that CAFs-Exo had a stronger ability to promote OSCC proliferation and was associated with immunosuppression. We discovered that the presence of immune-related genes in CAFs-Exo may regulate the expression of PIGR, CD81, UACA, and PTTG1IP in Cal-27 by analyzing CAFs-Exo sequencing data and publicly available TCGA data. This may account for the ability of CAFs-Exo to exert immunomodulation and promote OSCC proliferation.

**Conclusions:**

CAFs-Exo was found to be involved in tumor immune regulation through hsa-miR-139-5p, ACTR2 and EIF6, while PIGR, CD81, UACA and PTTG1IP may be potentially effective targets for the treatment of OSCC in the future.

**Supplementary Information:**

The online version contains supplementary material available at 10.1186/s12885-023-11028-5.

## Introduction

Oral squamous cell carcinoma (OSCC) is the most common malignancy among head and neck squamous cell carcinomas [1, 2]. Globally, OSCC accounts for 377,713 new diagnoses and 177,757 deaths, approximately 2% of all malignancies and 1.8% of cancer deaths in 2020 [3]. Although a comprehensive and multidisciplinary sequential treatment plan for OSCC has been developed, the 5-year survival rate has remained roughly 50 percent and has not significantly increased [4, 5]. Therefore, the key to reducing morbidity and mortality as well as enhancing patients' quality of life is the discovery of early diagnostic methods and targeted therapeutic strategies for OSCC.

Carcinoma-associated fibroblasts (CAFs) are permanently activated fibroblasts with potent tumor-modulating effects. By secreting platelet derived growth factor-BB (PDGF-BB), human oral mucosal fibroblasts (hOMF) are reprogrammed into CAFs by OSCC, and activated CAFs have significant tumorigenic effects [6, 7]. The material and information exchange between OSCC cells is mediated by CAFs-derived exosomes (CAFs-Exo), which are an important pathway for cell–cell interactions [8, 9].

Exosomes contain various components such as DNA, RNA, lipids, metabolites, cytoplasm and cell surface proteins [10]. Exosomes facilitate transcriptional-translational regulation and intercellular communication by serving as carriers for various nucleic acids or metabolites [11]. One of the characteristics of activated CAFs is the dysregulation of mRNAs and miRNAs, which leads to changes in the composition of CAFs-Exo as well and induces tumor resistance, invasion, and growth [12–14]. Previous studies have reported that miRNAs in CAFs-Exo promote proliferation and metastasis of OSCC [15, 16]. However, no study has reported how PDGF-BB-induced CAFs regulate the progression of OSCC after the release of CAFs-Exo.

Previous studies have shown that exosome-mediated immune regulation is one of the important mechanisms by which CAFs promote cancer progression [17, 18]. CAFs-Exo can influence the immune status of tumors by interacting with immune cells and key components of the tumor microenvironment [19]. It has been shown that the delivery of miRNAs in exosomes disrupts metabolic and immune responses, ultimately leading to cancer progression and metastasis [12]. As a result, we concentrated on the differences between CAFs-Exo and hOMF-derived exosomes (hOMF-Exo) components, especially in immune regulation. Additionally, we also used mRNA-miRNA interaction network analysis to reveal the possible interactions and regulation of mRNAs and miRNAs in CAFs-Exo with OSCC in immunomodulation. This provides new ideas and approaches to understand the biological mechanisms by which CAFs promote OSCC progression, and also lays the foundation for finding therapeutic targets for OSCC. The workflow of this study is shown in the Supplementary Fig. 1.

## Methods

### Induction of CAFs and extraction of CAFs-Exo

hOMF (CellResearchCorp, Singapore) were cultured in high glucose DMEM supplemented with 10% fetal bovine serum (Gibco, USA). The hOMF was treated with 30 ng/ml of PDGF-BB (Peprotech, UK) for 72 h according to previous conditions [6] and will be induced into CAFs. CAFs and hOMF were washed 3 times with PBS and incubated with Exo-ClearTM Complete Cell Growth Medium (SBI, USA) for 48 h. Supernatants of both cells were concentrated at 5,000 g for 30 min at 4 °C using 3 kDa ultrafiltration tubes (Millipore, USA), and then exosomes were extracted by exoEasy Maxi Kit (Qiagen, Germany) according to the manufacturer's protocol. Total protein was extracted from CAFs-Exo and hOMF-Exo and quantified using the BCA Protein Assay Kit (Beyotime, China). Exosomes were fixed with 4% paraformaldehyde for 10 min, dropped onto copper grids, stained with 1% uranyl acetate for 10 min, and observed in a transmission electron microscope (JEM-1011, Japan). The extracted exosomes were diluted to 3 × 10^7^—3 × 10^9^ / ml and the particle size was measured using Flow NanoAnalyzer (Flow Bio, China).

### Cal-27 cells were co-cultured with CAFs-Exo or hOMF-Exo

To clarify the uptake of exosomes in oral squamous cell carcinoma, Cal-27 (Cellcook, Guangzhou, China) was co-cultured with CAFs-Exo and hOMF-Exo. PKH26 (Sigma, USA) was used to label hOMF-Exo and CAFs-Exo according to the instructions. Cal-27 cells were seeded in a confocal culture dish, the labeled hOMF-Exo and CAFs-Exo were added to the culture dish and incubated for 6 h and 24 h. 4% paraformaldehyde was fixed and then rinsed in PBS. After the nuclei were stained with DAPI, images were acquired under a laser scanning confocal microscope (Nikon, Japan) in a wet state. The proliferation and cytotoxicity of Cal-27 cells were detected by cell counting kit (CCK-8, Beyotime, China). Cal-27 were seeded in 96-well plates at a density of 1.5 × 10^4^ cells/well. The absorbances were measured at 450 nm in a microplate reader and the cell proliferation rate was calculated using a standard curve based on the OD value at 450 nm. A wound-healing assay was applied to detect the migratory capability of Cal-27 cells. Culture-Insert was attached to the center of the 24-well plate. Cal-27 were incubated overnight in two chambers of Culture-Insert with 100 μl of cell suspension. Culture-Insert was gently removed the next day and culture medium containing hOMF-Exo and CAFs-Exo was added in groups and incubated at 37 °C in a 5% CO_2_ incubator. The gaps were observed and photographed at 0 h, 6 h,12 h and 24 h, the area of scratches were calculated by Image J software (Version 2).

### Tumor-forming and exosomes injection in nude mice

BALB/c-Nude mice were purchased from the Animal Experiment Center of Kunming Medical University. Mice were housed under standard conditions (12 h of light and 12 h of darkness, temperature 18–22 °C, humidity 55 ± 5%). All experimental protocols were approved by the Animal Experimentation Ethics Review Committee of Kunming Medical University (NO. KMMU2021750) and conducted in accordance with the Animal Research: Reporting of In Vivo Experiments (ARRIVE) guidelines. Fifteen half-male and half-female mice were randomly divided into three groups: five in the normal control (NC) group, five in the hOMF-Exo group, and five in the CAFs-Exo group. 2 × 10^6^ Cal-27 cells were subcutaneously injected into the nude mice (n = 5 each group). Exosomes or PBS were injected every 3 d around the tumor using a microfuge for a total of 6 injections at 100 μg CAFs-Exo, 100 μg hOMF-Exo or 100 μl PBS. On day 3 after the last injection, the mice were sacrificed by cervical dislocation. Animal euthanasia is conducted with reference to the American Veterinary Medical Association (AVMA) Guidelines for the Euthanasia of Animals (2020). The formation and growth of the transplanted tumors were observed, and the tumor sizes were recorded. The fixed tumor tissues was embedded in paraffin after gradient dehydration. Paraffin-embedded tissue slices, 10 μm in thickness. Then, a hematoxylin–eosin (HE) staining kit (Solarbio, Beijing, China) was used for HE staining. A portion of paraffin sections were left for immunohistochemical staining. After incubation with EDTA buffer (pH 8.0) to repair antigen (95–100 °C for 20 min), the sections were incubated with anti-CD3 or anti-CD8 in 1% BSA for 1 h at room temperature. Diaminobenzidine color development and hematoxylin counterstaining were performed, coverslips were subjected to ethanol dehydration, xylene transparency, and mounting. All glass slides were digitally scanned and semiquantitative analysis was performed using Image J software (Version 2).

### Protein extraction and western blotting

CAFs-Exo total protein, hOMF-Exo total protein and Cal-27 total protein were extracted using RIPA buffer with PMSF (Beyotime, China). Protein concentration was quantified by BCA protein assay (Beyotime, China). The protein samples were subjected to SDS/PAGE electrophoresis and transferred on to PVDF membranes. After blocked with 5% skim milk, the blots were probed with primary antibodies. Primary antibodies included CD9 (ab92726, Abcam, UK), CD63 (510953, Zeneng, China), CD81 (ab125011, Abcam, UK), HSP90 (TA500494, ORIGENE, USA), TSG101 (ab125011, Abcam, UK), β-microtubulin (M20005. Abmart, China), fibroblast activator protein-α (FAP, 506349, Zeneng, China), α-smooth muscle actin (α-SMA, bsm-33178 M, BIOSYNTHESIS, China). After incubation with the appropriate horseradish peroxidase-conjugated secondary antibody in TBST at room temperature for 1 h, enhanced chemiluminescence reagents (Beyotime, China) were used to detect interactions. Blots were imaged with the Chemidoc Imager (BioRad, USA) and semiquantitative analysis was performed using Image J software (Version 2).

### Detection of Cal-27 and exosome genes with quantitative PCR assay

Total RNA was extracted using Total RNA Isolation Kit (TIANGEN, China) following the protocol provided by the manufacturer. Small RNAs were reverse transcribed using the miRcute miRNA first-strand cDNA synthesis kit (TIANGEN, China). The mRNA template was reversely transcribed into cDNA using reverse transcriptase Kit (TIANGEN, China). Quantitative real‐time PCR (RT-qPCR) were conducted using the SuperReal PreMix Plus SYBR Green (Tiangen, China) on QuantStudio™ Real-Time PCR System (Thermo Fisher Scientific, USA). The 2^−ΔΔCT^ method was used to calculate the relative gene expression. The primers were synthesized by Sangon Biotech, Co., Ltd (Supplementary Table 1).

### Sequencing and raw data processing

The miRNAs and mRNA of hOMF-Exo and CAFs-Exo (three replicates for each group) were extracted and sequenced with DNBseq platform, with an average yield of 35.14 Mdata per sample. The average alignment ratio of the sample comparison genome was 98.68%. Differentially expressed miRNAs and mRNA were screened with the DEGseq package of R-Studio (Version 2022.11.0). The *t* test was used to identify miRNAs that were differentially expressed between CAFs and hOMF. The screening criteria were |fold change|≥ 1.5 and FDR < 0.05.

The total RNA of Cal-27 cells treated with PBS, hOMF-Exo or CAFs-Exo (three replicates for each group), were extracted and sequenced with BGISEQ-500 platform, averagely generating about 6.60G Gb bases per sample. HISAT was used to align the clean reads to the reference genome. Bowtie 2 to align the clean reads to the reference genes. Differentially expressed mRNA were screened with the DEGseq package of R-Studio (Version 2022.11.0).

### Identification of immune-associated genes

A comprehensive method to identify potential biomarkers in OSCC exosomes was developed by integrating gene expression data. For this purpose, we first built a matrice (expression) presented as mRNA/miRNAs (row) by sample (column). RNA-seq raw read counts were converted to TPM values to scale all comparable variates and normalised across all samples. The *t* test was used to identify genes that were differentially expressed between CAFs and hOMF. Then, genes were obtained based on *P*-values (*P* < 0.05). The *t* test *P-*values were retained for subsequent analyses. Finally, the differentially expressed genes (DEGs) were labeled with immune genes. Here, the immune genes come from the GO database and include all the genes in the immune-related terms.

### PPI network construction and module analysis

Search Tool for the Retrieval of Interacting Genes (STRING; version 11.5; http://string.embl.de/) is a database designed to construct a PPI network based on known and predicted PPI, and then the functional interactions between protein were analyzed. Based on the STRING online tool, the PPI of immune-related genes was constructed. Next, Cytoscape (version 3.9.1) was used to examine the potential correlation between these immune-related genes. At the same time, the MCODE application in Cytoscape was used to check the modules of the PPI network (degree cutoff = 3, maximum depth = 50, k core = 2, node score cutoff = 0.2).

### Screening of candidate genes related to immune cells

In order to compare the differences in immune cell subtypes, based on immune-related gene expression files, the CIBERSORT package (version 1.03) was used to evaluate the proportion of 22 immune cell subtypes using LM22 signatures and 1000 permutations. Cases with a CIBERSORT output of *P* < 0.05 were eligible for further analysis. Mann–Whitney U test was used to compare differences in immune cell subtypes in the CAFs and hOMF groups.

Our aim was to capture genes with relatively high co-expression in immune cells. To implement this, the first step is the normalization between the datasets using Scale function in R software. Then, we define the correlation between gene expression and each immune cell score (g_i_, c_j_) in the dataset, referred to as S_ij_:

$${\mathrm S}_{\mathrm{ij}}=\left\{\left.Pcor\left(g_i,c_j\right)\right|\mathrm i,\mathrm l\leq\mathrm j\leq22\right\}$$

where Pcor (g_i_, c_j_) is the Pearson correlation significance *P-*values between the genes g_i_ and immune cells c_j_.

Each immune cell-related gene set was matched with the gene set of the PPI module to obtain candidate genes related to each immune cell.

### mRNA-miRNA interaction network construction by WGCNA

We obtained differentially expressed miRNAs (the method is the same as DEGs), and used the R package named weighted gene co-expression network analysis (WGCNA; version 1.6.9) to construct an immune-related gene and differentially expressed miRNAs co-expression network. Firstly, Pearson correlation was used to evaluate the weighted interaction relationship between subjects in all data sets in the adjacency matrix, and abnormal samples were removed to ensure the reliability of the network construction results. Here, cutHeight was set to 7, and minSize was set to 10. Secondly, the soft-thresholding power of network construction was selected so that the constructed network accords with the power-law distribution and was closer to the real biological network state. Since the sample size was less than 20, the soft-thresholding power was determined to be 9. Then, the weighted adjacency matrix is transformed into a topological overlap measure matrix, and its connectivity in the network is estimated. The average link hierarchical clustering method is used to construct the clustering dendrogram of the topological overlap matrix (TOM). Set the smallest gene module size to 30 to obtain a suitable module, and set the threshold for merging similar modules to 0.25.

### Development of immune-related signature

The set including 369 OSCC cases were downloaded from the TCGA database. Tumor sites of oral cavity, oral tongue, buccal mucosa, lip, alveolar ridge, hard palate, and floor of mouth were included. First, we defined the sample whose age was less than or equal to 50 years old as middle younger, the sample between 50 and 75 years old as quinquagenarian, and the age greater than or equal to 75 years old as agedness. To further clarify the relationship between candidate genes expression and OSCC prognosis, the OS between different groups was compared by Kaplan–Meier analysis with the log-rank test.

Here, we only focused on the immune module with the most candidate genes related to immune cells. Then, the top 30 miRNAs with the highest TOM similarity of each candidate gene in the immune module were screened, among which the miRNAs belonging to the immune module constituted the miRNAs set of candidate gene. The miRNAs that are common in all miRNAs sets are called target miRNAs. A prognostic signature was constructed using the linear combination of the expression values of the target miRNAs and their candidate genes, weighted by their estimated regression coefficients in the multivariate Cox regression analysis.

### Analysis of CAFs-Exo target genes by mRNA-miRNA interaction network

To investigate the target genes of CAFs-Exo in Cal-27, we analyzed the DEGs of Cal-27 after co-culture with CAFs-Exo or hOMF-Exo. In combination with TargetScan (version 8.0; https://www.targetscan.org), miRanda (version 3.3a; https://www.microrna.org) and RNAhybrid databases (version2.1.2; https://bibiserv.cebitec.uni-bielefeld.de/rnahybrid), overlapping genes were used for mRNA-miRNA interaction network analysis and the screened target genes were considered to be associated with CAFs-Exo. The acquired target genes were used for survival analysis. The target genes were validated by RT-qPCR in cells and tumors.

### Statistical analysis

The GraphPad Prism software (version 9.0) was used for data analysis and visualization. The data provided herein are expressed as the mean ± standard deviation (SD) of at least three experimental replicates. The normality of the data was judged using the Shapiro–Wilk test. One way analysis of variance (ANOVA) was used to compare the overall mean difference between multiple groups of data, and statistically different data were then tested with least significant difference (LSD) test for differences between the two groups. For data that did not conform to a normal distribution, we used the Kruskal–Wallis test to compare the statistical significance of the groups. *P* < 0.05 indicated the difference to be statistically significant.

## Results

### PDGF-BB induces hOMF reprogramming to CAFs and CAFs-Exo release

**Fig. 1:**
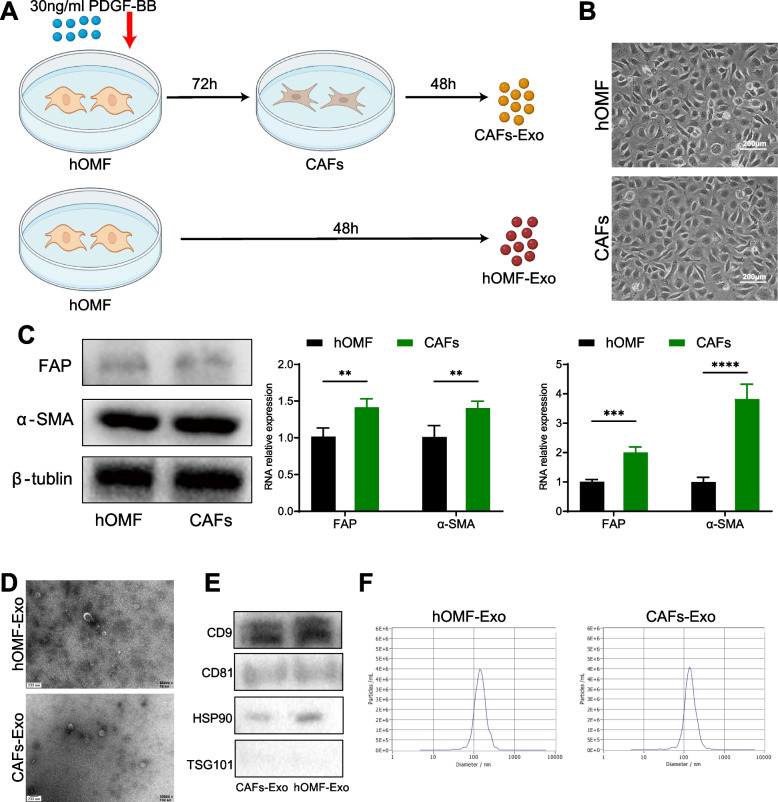
Induction of CAFs and identification of exosomes. **A** Reprogram hOMF to CAFs. **B** The changes in cell shape were observed under an inverted microscope after reprogramming. **C** Detection of protein and mRNA expression of FAP and α-SMA in hOMF and CAFs. **D** CAFs-Exo and hOMF-Exo under transmission electron microscopy. **E** The expression of CD9, CD81, HSP90 and TSG101 in CAFs-Exo and hOMF-Exo was detected by Western blotting. **F** The particle size of CAFs-Exo and hOMF-Exo was analyzed using Flow nanoanalyzer. ** *P* < 0.01; *** *P* < 0.001

Our previous study demonstrated that PDGF-BB induces reprogramming of hOMF to CAFs [6]. Using previous conditions (Fig. 1A), after 72 h of stimulation in PDGF-BB-containing cultures, hOMF showed significantly higher mRNA and protein levels of the CAFs markers SMA and FAP. The results prompted hOMF reprogramming to CAFs (Fig. 1B-C). Extraction of exosomes from cell culture supernatants using ultrafiltration, including CAFs-Exo and hOMF-Exo derived from CAFs and hOMF. Both CAFs-Exo and hOMF-Exo showed round vesicle-like structures with bilayer membranes and similar particle sizes under transmission electron microscopy (Fig. 1D). Expression of exosomal markers was detected by western blotting, and CD9, CD81, HSP90 and TSG101 were expressed in both CAFs-Exo and hOMF-Exo (Fig. 1E). The results of flow nanoanalysis showed homogeneous particle size for both CAFs-Exo and hOMF-Exo. The average particle size of CAFs-Exo was 69.25 nm and the average particle size of hOMF-Exo was 70.75 nm (Fig. 1F).

### CAFs-Exo are taken up by Cal-27 and promote cell proliferation and migration

**Fig. 2:**
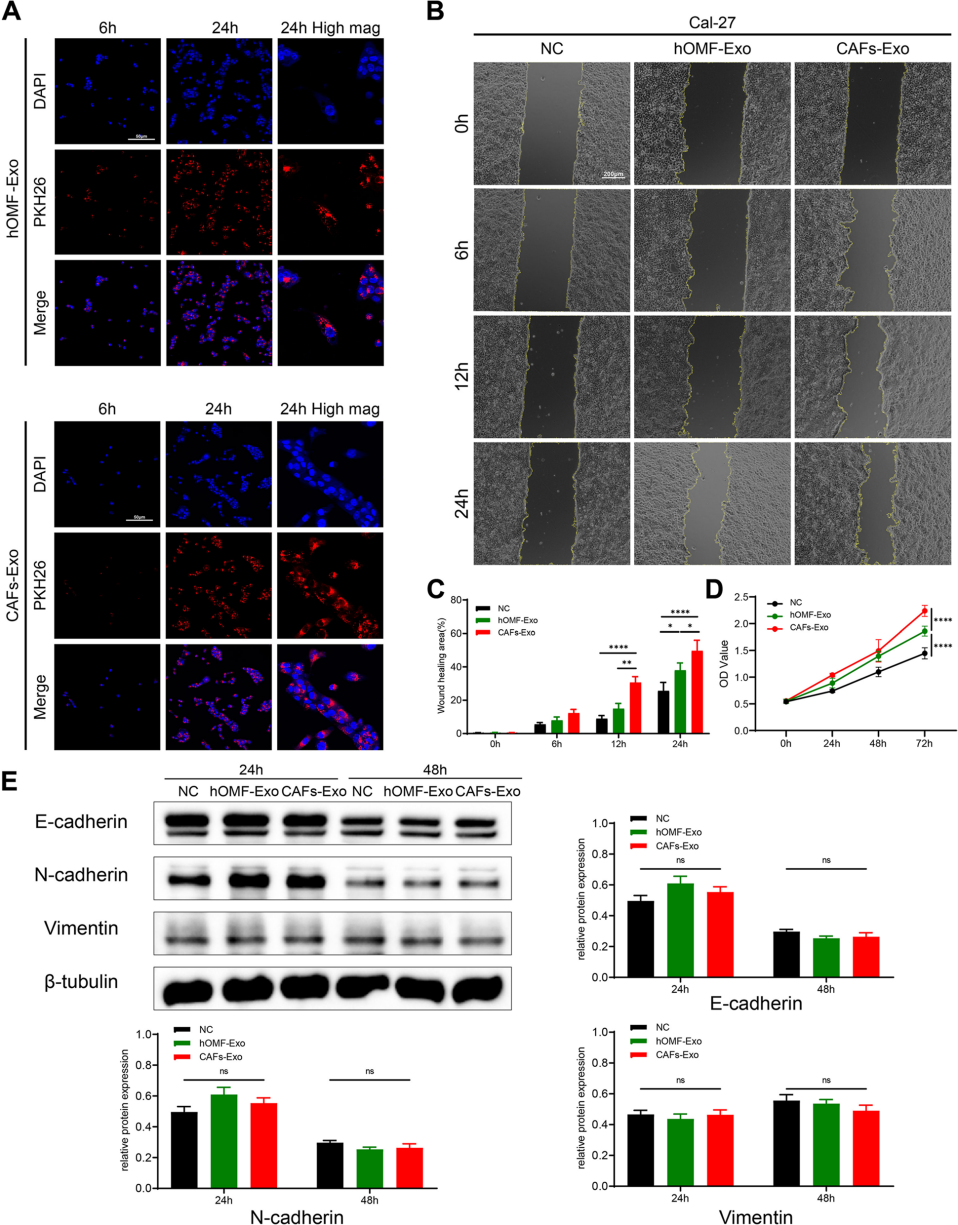
Exosomes are taken up by Cal-27 and promote cell proliferation and migration. **A** After labelled with PKH2, hOMF-Exo and CAFs-Exo were co-cultured with Cal-27 cells for 6 h and 24 h, and observed under Laser Scanning Confocal Microscope. **B** Exosomes co-cultured with Cal-27 cells for 0 h, 6 h, 12 h and 24 h to observe wound healing under microscope. **C** The wound area results showed that exosomes co‐cultured with Cal-27 cells could promote migration ability. **D** CCK8 assay indicated that exosomes promote the proliferation of Cal-27. **E** The expression of N-cadherin, E-cadherin and Vimentin did not differ significantly between the groups. * *P* < 0.05; ** *P* < 0.01; **** *P* < 0.0001; *ns*, no significance* P* > 0.05

To clarify whether the exosomes were taken up by oral squamous cell carcinoma, PKH26-labeled hOMF-Exo or CAFs-Exo were co-cultured with Cal-27. After 6 h of co-culture, a small amount of exosomes accumulated around the nucleus of Cal-27 cells, and the amount of aggregation increased significantly after 24 h. The results indicated that at 6 h, Cal-27 had started to take up exosomes and the amount of uptake increased with time (Fig. 2A). In this study, the effect of exosomes on Cal-27 migration ability was observed by wound-healing assay (Fig. 2B-C). The results showed that at 24 h, the migration rate of Cal-27 was significantly faster with the addition of CAFs-Exo co-culture (hOMF-Exo *vs* CAFs-Exo, *P* = 0.0346; CAFs-Exo *vs* NC, *P* < 0.0001; hOMF-Exo *vs* NC, *P* = 0.0253). Using a cell co-culture system with hOMF-Exo/CAFs-Exo, the cell proliferation of Cal-27 was examined. The results showed that CAFs-Exo promoted Cal-27 cell proliferation most significantly at 72 h (CAFs-Exo *vs* NC, *P* < 0.0001; hOMF-Exo *vs* NC, *P* < 0.0001), indicating that CAFs-Exo promotes the proliferation of Cal-27 (Fig. 2D). To determine whether the difference in wound-healing is due to epithelial-mesenchymal transition (EMT), we detected the expression of N-cadherin, E-cadherin and Vimentin (Fig. 2E). The results showed that at 24 h and 48 h, the markers were expressed indistinguishably between groups, indicating that the migration changes were not affected by EMT.

### CAFs-Exo promote the tumor formation by Cal-27 in vivo and is associated with immune modulation

**Fig. 3:**
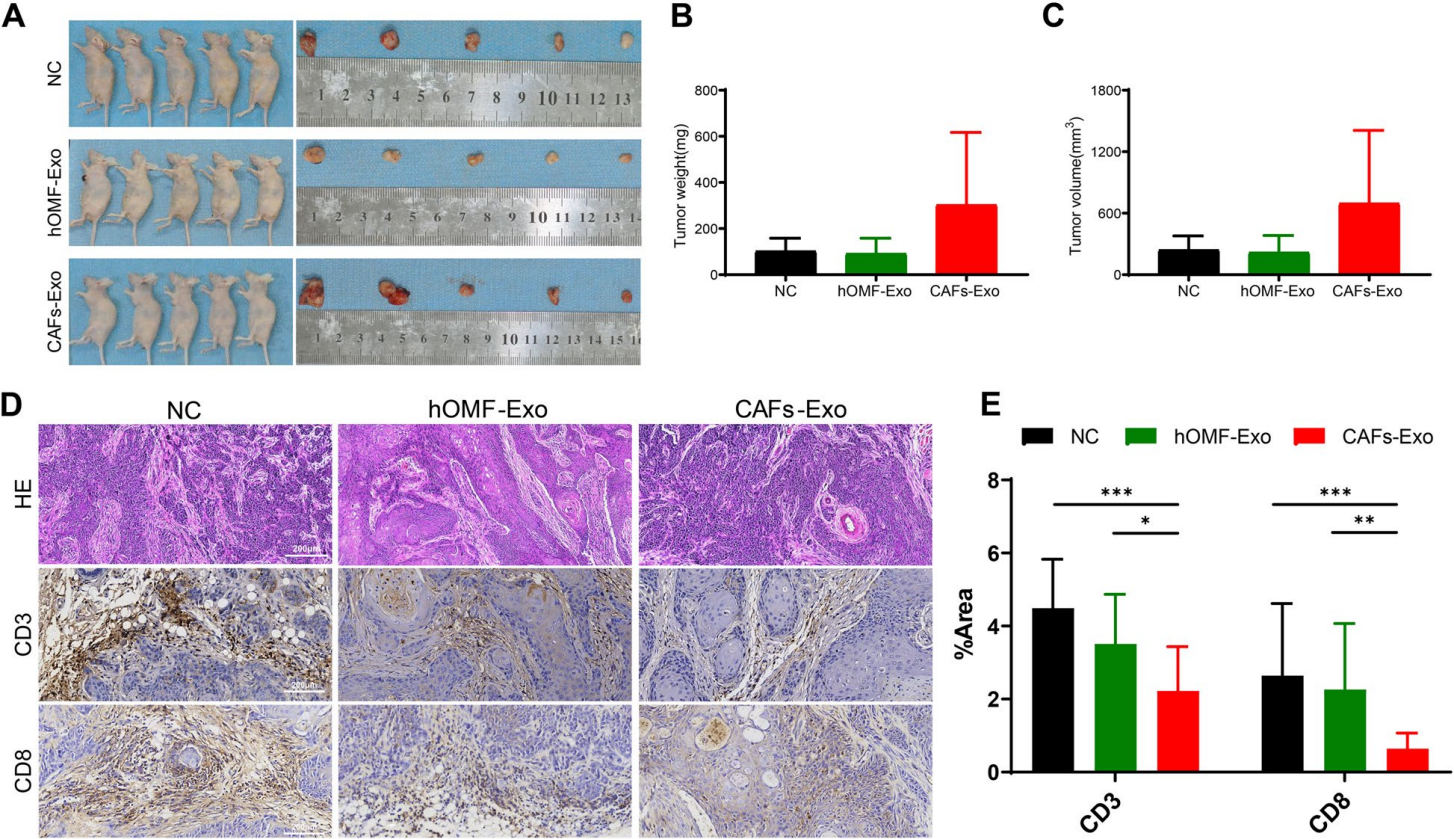
Exosomes promote Cal-27 tumor growth and correlate with immune regulation. **A** The tumors were measured and weighted after 21 days of inoculation of the mixture of Cal-27 cells and hOMF-Exo or CAFs-Exo in BALB/c-Nude mice. **B-C** Tumor weight and volume statistics results. **D** The analysis of tumor by HE staining, and CD3 and CD8 labelling. **E** Immunohistochemical staining statistics. * *P* < 0.05; ** *P* < 0.01; *** *P* < 0.001

Cal-27 was inoculated subcutaneously into nude mice and exosomes or PBS was injected next to the tumor with a microinjector. Tumor growth was visible in about 80% of nude mice after 1 week, and obvious tumors were visible in all groups after 2 weeks. Mice were sacrificed 21 days after inoculation with Cal-27 cells, and tumor tissues were taken, photographed, weighed and measured (Fig. 3A-C). In the CAFs-Exo group, three nude mice had subcutaneous tumors invading the chest wall with severe adhesions, and two of the ribs could not be completely separated from the tumors. The tumor weight (NC *vs* CAFs-Exo, *P* = 0.2294; hOMF-Exo *vs* CAFs-Exo,* P* = 0.2095) and total volume (NC *vs* CAFs-Exo, *P* = 0.2295; hOMF-Exo *vs* CAFs-Exo, *P* = 0.2108) were greater in the CAFs-Exo group than in the NC and hOMF-Exo groups. To clarify whether tumor proliferation was associated with the immune microenvironment, we sectioned and immunohistochemically stained tumor tissues. HE sections showed highly differentiated squamous cell carcinomas in all groups (Fig. 3D). Positive CD3 and CD8 indicate excess cellular immunity [20–22]. Immunohistochemical results showed that CD3 and CD8 positive cells were significantly reduced in the CAFs group and showed high expression in the NC and hOMF-Exo groups (Fig. 3E). These results suggest that CAFs-Exo may lead to reduced immune cell infiltration in transplanted tumors and increase the proliferative capacity of Cal-27.

### CAFs-Exo is associated with multiple immune regulatory genes

**Fig. 4:**
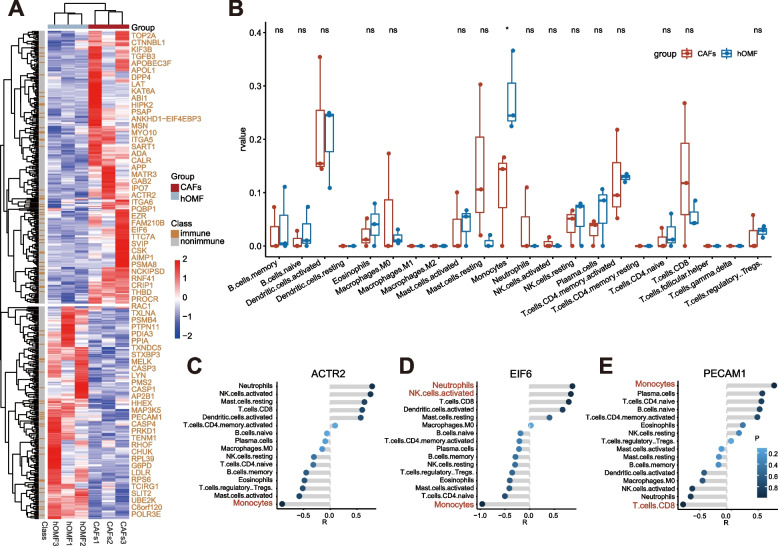
Dysregulated mRNAs reveal several significant immune cells. **A** Heat map of differentially expressed mRNAs between CAFs-Exo and hOMF-Exo. **B** The difference of tumor-infiltrating immune cells among two groups. **C-E** Histogram of correlation coefficients and significant *P*-values between candidate genes and immune cells. *: *P* < 0.05; *ns*: no significance

To investigate the mechanism of CAFs-Exo promoting tumor growth, we first sequenced the mRNAs of CAFs-Exo and hOMF-Exo. The results showed that 473 mRNAs were differentially expressed, of which 267 mRNAs were up-regulated and 206 mRNAs were down-regulated. Further analysis revealed that 70 mRNAs were associated with immunity (Fig. 4A). The CIBERSORT tool was used to estimate the presence of candidate genes and types associated with immune cells among the 70 immune-related differential mRNAs. The results revealed enrichment of 16 out of 22 immune cell types, with monocyte infiltration showing relatively high abundance and significant differences in CAFs-Exo and hOMF-Exo (*P* < 0.05) (Fig. 4B). A PPI network consisting of 70 nodes (immune-related mRNAs) and 169 edges (interactions) was constructed using STRING (Supplementary Fig. 2A). Then six modules were mined by OH-PIN, IPCA, EAGLE, and MCOMD algorithms in the cytoscape tool (Supplementary Fig. 2B-G), suggesting that these modules may play an important role in the development of OSCC.

We analyzed the correlation between the infiltration score of immune cells and mRNA expression profiles, and we found that 11 mRNAs (ACTR2, CRIP1, EIF6, FAM210B, HHEX, IPO7, PDIA3, PECAM1, PPIA, SVIP, and TXNDC5) were associated with monocyte infiltration. Among them, ACTR2, EIF6, and PECAM1 overlapped with mRNAs in the PPI module. Further analysis showed that ACTR2, EIF6, and PECAM1 were significantly associated with infiltration of at least one immune cell type (*P* < 0.05, |R|> 0.5) (Fig. 4C-E). Therefore, ACTR2, EIF6, and PECAM1 are considered to be candidate genes associated with immune regulation in exosomes.

### Co-expression network of exosomal mRNA-miRNA constructed by WGCNA analysis

We identified the differential miRNAs of CAFs-Exo and hOMF-Exo by high-throughput sequencing. A total of 801 miRNAs were detected. When |fold change|> 1, *P* < 0.05, there were 162 differential miRNAs between CAFs-Exo and hOMF-Exo. We used WGCNA to analyze the exosomal mRNA and miRNA sequencing results and construct an mRNA-miRNA co-expression network. When the soft-thresholding power in WGCNA was determined as 9, three modules were identified based on the average linkage hierarchical clustering and soft threshold power (Figure S3A-C). Among all modules, the blue module is considered as the immune-related module and contains 29 mRNAs and 36 miRNAs. Two candidate mRNAs, ACTR2 and EIF6, were included in the blue module.

### ACTR2, EIF6 and hsa-miR-139-5p are associated with survival for OSCC

**Fig. 5:**
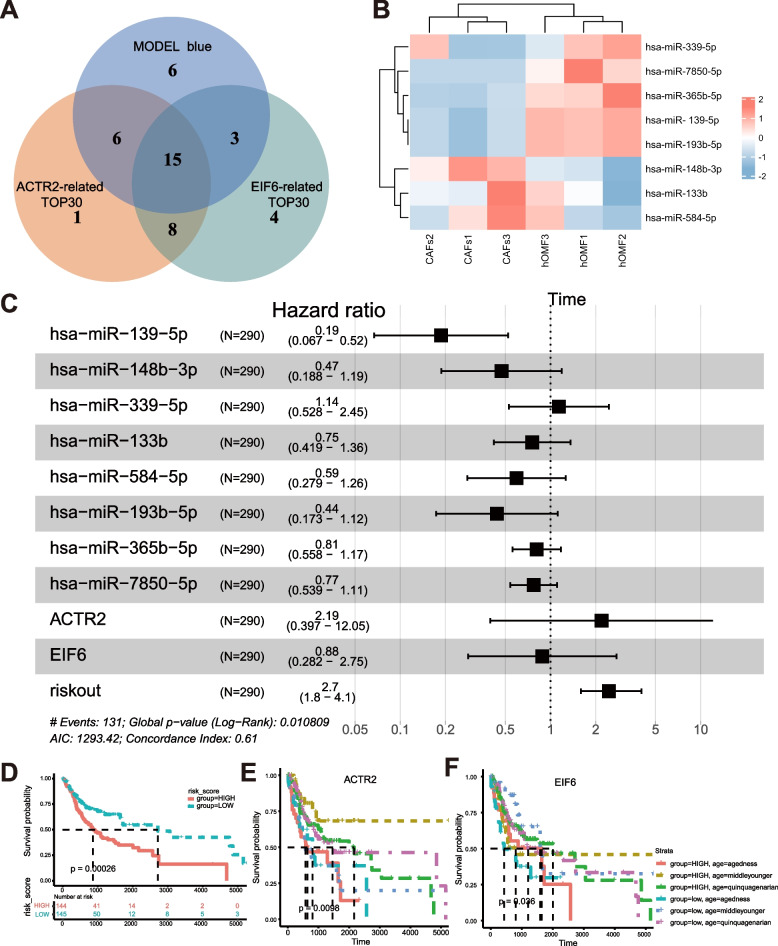
Candidate mRNAs and miRNAs as biomarkers of OSCC. **A** Venn diagram of shared miRNAs between model blue, the 30 most relevant miRNAs for ACTR2 and EIF6. **B** Heat map analysis of 8 miRNAs. **C** Forest plots of the results of multivariate Cox regression analyses of significant prognostic factors. **D** Kaplan–Meier survival analysis of the risk score. **E–F** Kaplan–Meier survival analysis of candidate genes in TCGA

A dataset of 369 OSCC cases was downloaded from the TCGA data portal. Pearson correlation analysis was used in these datasets to identify the 30 most relevant miRNAs for ACTR2, EIF6. The miRNAs in the immune-related module were intersected with the 30 most relevant miRNAs of ACTR2 and EIF6, and there were 15 overlapping miRNAs in total (Fig. 5A). Among the 15 miRNAs with exosome sequencing results, 8 miRNAs with expression data deletion rates below 60% were hsa-miR-139-5p, hsa-miR-148b-3p, hsa-miR-339-5p, hsa-miR-133b, hsa-miR-584-5p, hsa-miR-193b-5p, hsa-miR-365b-5p, and hsa-miR-7850-5p. Heat mapping was performed for these 8 miRNAs (Fig. 5B). In a Cox regression analysis of 8 miRNAs showed that hsa-miR-139-5p was significantly associated with cancer survival as a protective factor (Fig. 5C, HR = 0.19, *P* = 0.001). Risk scores were calculated for patients in the dataset, and the median (-0.123) was used as the cutoff value. Patients with higher risk scores had significantly shorter overall survival (OS) times compared to those with lower risk scores (Fig. 5D). Moreover, the prognostic significance of the 2 candidate mRNAs ACTR2 and EIF6 mentioned above was supported in this cohort (Fig. 5E-F). It means that ACTR2, EIF6 and hsa-miR-139-5p may be the key candidate factors in the immunoregulation capability of CAFs-Exo.

### ACTR2, EIF6 and hsa-miR-139-5p in exosomes regulate multiple Cal-27 cellular target genes

**Fig. 6:**
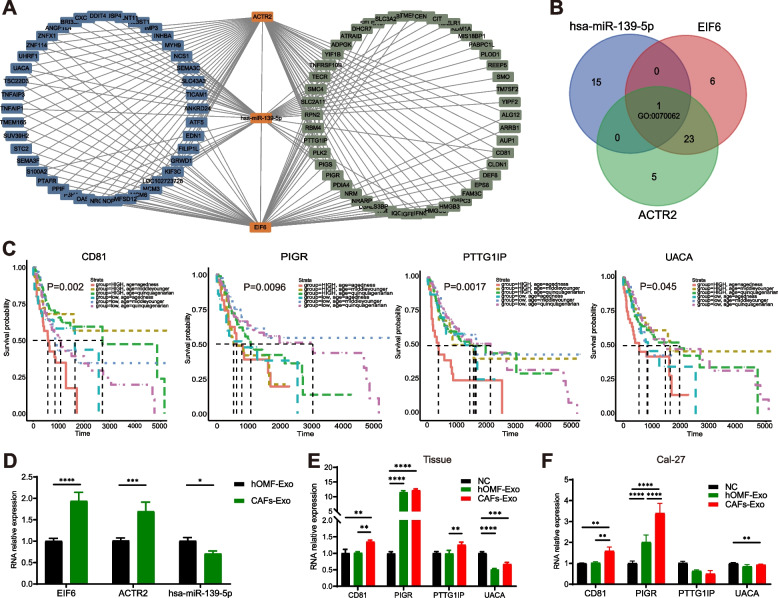
Validation of exosome-related mRNA-miRNA regulatory relationship. **A** The network shows the interaction among ACTR2, EIF6, hsa-miR-139-5p and D (blue), S (green) gene sets. **B** Venn diagram of shared GO terms among ACTR2, EIF6, hsa-miR-139-5p. **C** Kaplan–Meier survival analysis of four candidate genes in TCGA. **D** The candidate genes expression level in hOMF-Exo and CAFs-Exo. **E** PIGR, CD81, UACA and PTTG1IP expression level in tumor tissue. **F** PIGR, CD81, UACA and PTTG1IP expression level in Cal-27 cells. * *P* < 0.05; ** *P* < 0.01; *** *P* < 0.001; **** *P* < 0.0001

To investigate the potential target genes of exosomes in Cal-27 cells, CAFs-Exo or hOMF-Exo were used to co-culture with Cal-27 cells, and total RNA of Cal-27 cells was extracted for transcriptome sequencing. There were 222 DEGs after co-culture of CAFs-Exo with Cal-27 compared to the NC or hOMF-Exo groups (FDR < 0.05, fold change < -1). ACTR2, EIF6, and hsa-miR-139-5p were analyzed with 222 DEGs using the mRNA-miRNA interaction network. The interaction network demonstrated the scale-free topology of the transcriptional regulatory network, and consisted of 93 nodes (1 miRNA and 92 mRNAs) and 164 edges (Fig. 6A). Functional enrichment analysis was performed on the ACTR2, EIF6 and hsa-miR-139-5p relevant genes respectively. GO:0070062 is one common GO term in which ACTR2, EIF6, and hsa-miR-139-5p relevant genes enriched(Fig. 6B). There are 4 genes (UACA, PTTG1IP, PIGR, CD81) of this GO term may interacted with ACTR2, EIF6 and hsa-miR-139-5p at the same time, and they play an important role in cancer associated pathway: apoptotic process (UACA, PTTG1IP) and immunity (PIGR, CD81). Survival analysis of PIGR, CD81, UACA and PTTG1IP using a dataset of 369 OSCC cases showed that their expression was all significantly associated with OS (Fig. 6C).

We used RT-qPCR to examine the expression of relevant genes in Cal-27 cells, nude mouse tumor tissues, and exosomes to confirm the bioinformatic analysis's findings. The results showed that hsa-miR-139-5p was highly expressed in hOMF-Exo (*P* = 0.046), and EIF6 (*P* < 0.0001) and ACTR2 (*P* < 0.0001) were highly expressed in CAFs-Exo (Fig. 6D). Compared to the hOMF-Exo group, in the tumor tissues of nude mice after CAFs-Exo injection (*P* = 0.0015) and in Cal-27 cells co-cultured with CAFs-Exo (*P* = 0.0035), CD81 expression was significantly elevated (Fig. 6E-F). In the tumor tissues of nude mice after CAFs-Exo injection (*P* < 0.0001) and in Cal-27 tumor cells co-cultured with CAFs-Exo (*P* < 0.0001), the expression of PIGR was significantly higher than that in the NC group. In Cal27 cells, the expression of PIGR was significantly higher in the CAFs-Exo group than in the hOMF-Exo group (*P* < 0.0001). Although the variability of PTTG1IP and UACA was smaller than that of CD81 and PIGR, the results were generally consistent with previous bioinformatic analyses. The above results suggest that CAFs-Exo targets and regulates oncogenes, which in turn decreases the level of immune cell infiltration in transplanted tumors and increases the growth of tumors in nude mice.

## Discussion

OSCC is a malignant tumor of the oral epithelium that is currently treated primarily with radiotherapy and surgical resection. The tumor microenvironment mainly consists of tumor cells, infiltrating immune cells, CAFs, endothelial cells, adipocytes and other signaling molecules together, which have an important role in regulating the development of OSCC [23, 24]. OSCC tissues contain up to 80% of CAFs, and hOMF is reprogrammed and activated in the tumor microenvironment to produce CAFs [25]. Activated CAFs undergo significant changes in morphological structure, growth pattern, proliferative activity, motility, and secretory function [26]. CAFs regulate the tumor microecosystem and promote OSCC development, progression, and metastasis [27]. It is essential to investigate the ways in which CAFs promote OSCC malignant behavior.

The previous studies have demonstrated that OSCC induces reprogramming of hOMF to CAFs through high secretion of PDGF-BB, and activated CAFs produce extracellular matrix and exosomes that promote OSCC growth [6]. The hOMF highly expresses CAFs markers such as α-SMA and FAP-α after PDGF-BB intervention, while the activated hOMF further promotes the massive secretion of PDGF-BB by cancer cells through refeeding, forming a positive feedback reciprocal loop [6]. In this study, we generated CAFs induced by PDGF-BB, and the obtained CAFs-Exo were able to be taken up by Cal-27 and promoted the proliferation and migration of Cal-27 cells. In tumor formation experiments in nude mice, we found that CAFs-Exo promoted tumor growth, resulting in a significant decrease in the number of CD3 + and CD8 + cells. It has been shown that the number of CD3 + and CD8 + cells is an important reference for determining the prognosis of OSCC, and changes in the number of these cells are closely associated with dysregulation of the immune microenvironment [20–22]. Therefore, the analysis of exosome sequencing data focused more on changes in immune module-related genes and possible immune regulatory pathways.

CAFs-Exo complete cell-to-cell communication through the exchange of miRNAs, mRNAs and lncRNAs, etc., thus contributing to the development of OSCC [28–30]. Immunomodulatory pathways may be a specific mechanism by which CAFs promote OSCC proliferation [31, 32]. In this study, we performed bioinformatic analysis using RNA sequencing data of exosomes and Cal-27, and identified candidate genes that are all immune-related. Significant differences in the expression of ACTR2, EIF6 and hsa-miR-139-5p in CAFs-Exo and hOMF-Exo, and their prognostic significance are supported in the TCGA dataset. ACTR2 has been shown to be a major component protein of the encoded ARP2/3 complex that promotes cancer migration and invasion, and it is associated with immune cell infiltration [33]. High expression of EIF6 activates epithelial-mesenchymal transition, promotes OSCC cell migration and invasion, and is a potential therapeutic target [34]. It was found that high expression of hsa-miR-139-5p inhibited the development of OSCC and was strongly associated with survival [35]. Meanwhile, hsa-miR-139-5p inhibited HOXA9 expression in Cal-27, thereby suppressing OSCC proliferation, invasion and migration [36]. All these findings imply that candidate genes may be involved in the specific regulatory mechanism of CAFs-Exo in OSCC.

Integrative mRNA-miRNA network analysis is a common bioinformatic screening method used to analyze target genes [37–39]. In this study, the mRNA-miRNA network interaction analysis was used to identify four potential target genes of CAFs-Exo, all of which play important roles in cancer development. Importantly, the regulation pattern of ACTR2, miR-139-5p to PIGR and CD81 is consistent with the expression regulation pattern of PIGR and CD81 in OSCC. Related studies reported that CD81 contributes to tumor growth, as well as tumor cell migration and invasion [40]. Elimination of CD81 inhibited tumor growth and metastasis by immunomodulation [41, 42]. PIGR has been reported to promote tumor growth and participate in immune disorders [43, 44]. In this study, CD81 and PIGR were highly expressed after CAFs-Exo intervention. We speculate that the upregulated ACTR2 and downregulated miR-139-5p in CAFs-Exo may upregulate PIGR and CD81, thereby inhibiting immune cell infiltration.

Our findings highlight that hsa-miR-139-5p and ACTR2 from CAFs-Exo may have upregulated the expression of CD81 and PIGR in the Cal-27. This may be the cause of CAFs-Exo's special OSCC proliferation-promoting ability. There were several limitations associated with the present study. Firstly, we have verified the expression of the candidate gene and the target gene. However, there is no direct evidence to prove the regulatory relationship of the candidate gene in CAFs-Exo. Secondly, CAFs-Exo obtained from the activation of hOMF by PDGF-BB can effectively interfere with the proliferation and migration of Cal-27, but other factors affecting Cal-27 such as soluble factor, apoptotic body and inflammatory body are worth further study. Thirdly, limited by the difficulty of accessing clinical specimens, our approach is to analyze the gene expression profiles of OSCC in the TCGA database to validate our results, and more mRNA and miRNA data from the same samples are still needed for validation in the future to provide more convincing results.

## Conclusions

This study shows possible crosstalk and regulation between CAFs-Exo and the immune system in OSCC. We found that miRNAs and mRNAs in CAFs-Exo play an important role in the prognosis of OSCC patients. We also reveal the potential regulatory mechanisms of CAFs-Exo released by PDGF-BB after stimulating hOMF for UACA, PTTG1IP, PIGR, CD81 in Cal-27. In the future, these results will require more data to derive and more in-vitro and in-vitro experimental validation.

## Supplementary Information

**Additional file 1: Supplementary Fig. 1.** Experimental technical roadmap. **Supplementary Fig. 2.** PPI network reveals several immune-related modules. (A) PPI network of differentially expressed immune-associated genes. (B-G) Six modules may play an important role in the development of OSCC. The redder the edge, the higher the co-expression, and the redder the dot, the higher the degree. ppi refers to protein interactions. **Supplementary Fig. 3.** Key module correlated with OSCC identified by WGCNA. (A) The heatmap depicts the TOM among all genes in the analysis. (B) Clustering of all modules. (C) Cluster dendrogram of genes. **Supplementary Table 1.** Primer sequences for RT-qPCR.

**Additional file 2.**

## Data Availability

The datasets supporting the conclusions of this article are available by contacting corresponding author (Email: youdingyun@qq.com). The Supplementary Material for this article can be found online. All sequencing data have been uploaded to the GEO database with the accession number Series GSE222278.

## Abbreviations

ANOVA: One way analysis of variance
ARRIVE: Animal Research: Reporting of In Vivo Experiments
AVMA: American Veterinary Medical Association
CAFs: Cancer-associated fibroblasts
CAFs-Exo: CAFs-derived exosomes
DEGs: Differentially expressed genes
EMT: Epithelial-mesenchymal transition
HE: Hematoxylin–eosin
hOMF: Human oral mucosa fibroblast
hOMF-Exo: HOMF-derived exosomes
LSD: Least significant difference
NC: Normal control
OS: Overall survival
OSCC: Oral squamous cell carcinoma
PDGF-BB: Platelet derived growth factor-BB
RT-qPCR: Quantitative real‐time PCR
SD: Standard deviation
TOM: Topological overlap matrix
WGCNA: Weighted gene co-expression network analysis
