# Supplementary material for: Analysis of mRNA-miRNA interaction network reveals the role of CAFs-derived exosomes in the immune regulation of oral squamous cell carcinoma
Source: BMC Cancer. 2023 Jun 26;23:591. doi: 10.1186/s12885-023-11028-5 (PMC10294399; doi:10.1186/s12885-023-11028-5)
Supplement: Supplementary file 1 — Additional file 1: Supplementary Fig. 1. Experimental technical roadmap. Supplementary Fig. 2. PPI network reveals several immune-related modules. (A) PPI network of differentially expressed immune-associated genes. (B-G) Six modules may play an important role in the development of OSCC. The redder the edge, the higher the co-expression, and the redder the dot, the higher the degree. ppi refers to protein interactions. Supplementary Fig. 3. Key module correlated with OSCC identified by WGCNA. (A) The heatmap depicts the TOM among all genes in the analysis. (B) Clustering of all modules. (C) Cluster dendrogram of genes. Supplementary Table 1. Primer sequences for RT-qPCR. [file 12885_2023_11028_MOESM1_ESM.pdf]

Supplementary Material

Analysis of mRNA-miRNA interaction network reveals the role of CAFs-derived exosomes in the immune regulation of oral squamous cell carcinoma

Wei-Zhou Wang†, Xue Cao†, Li Bian†, Gao Yue, Ming Yu, Yi-Ting Li, Jian-Guo Xu, Yang-Hao Wang, He-Feng Yang*, Ding-Yun You*, Yong-Wen He*

*** Correspondence:** Corresponding Author: email@uni.edu Yong-Wen He: heyongwen2@sina.com; Ding-Yun You: youdingyun@qq.com; He-Feng Yang: yanghefeng2008@163.com.

† These authors contributed equally to this work.

1 Supplementary Figures

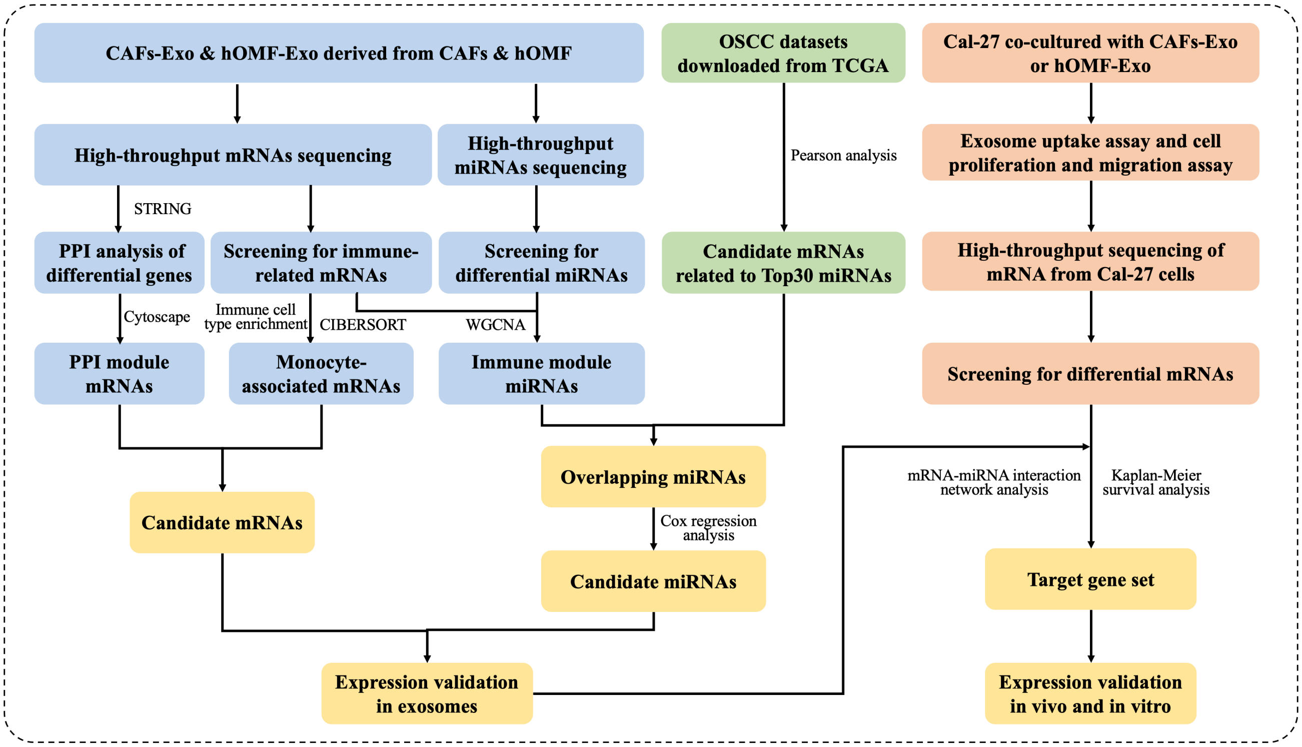

Supplementary Figure 1. Experimental technical roadmap.

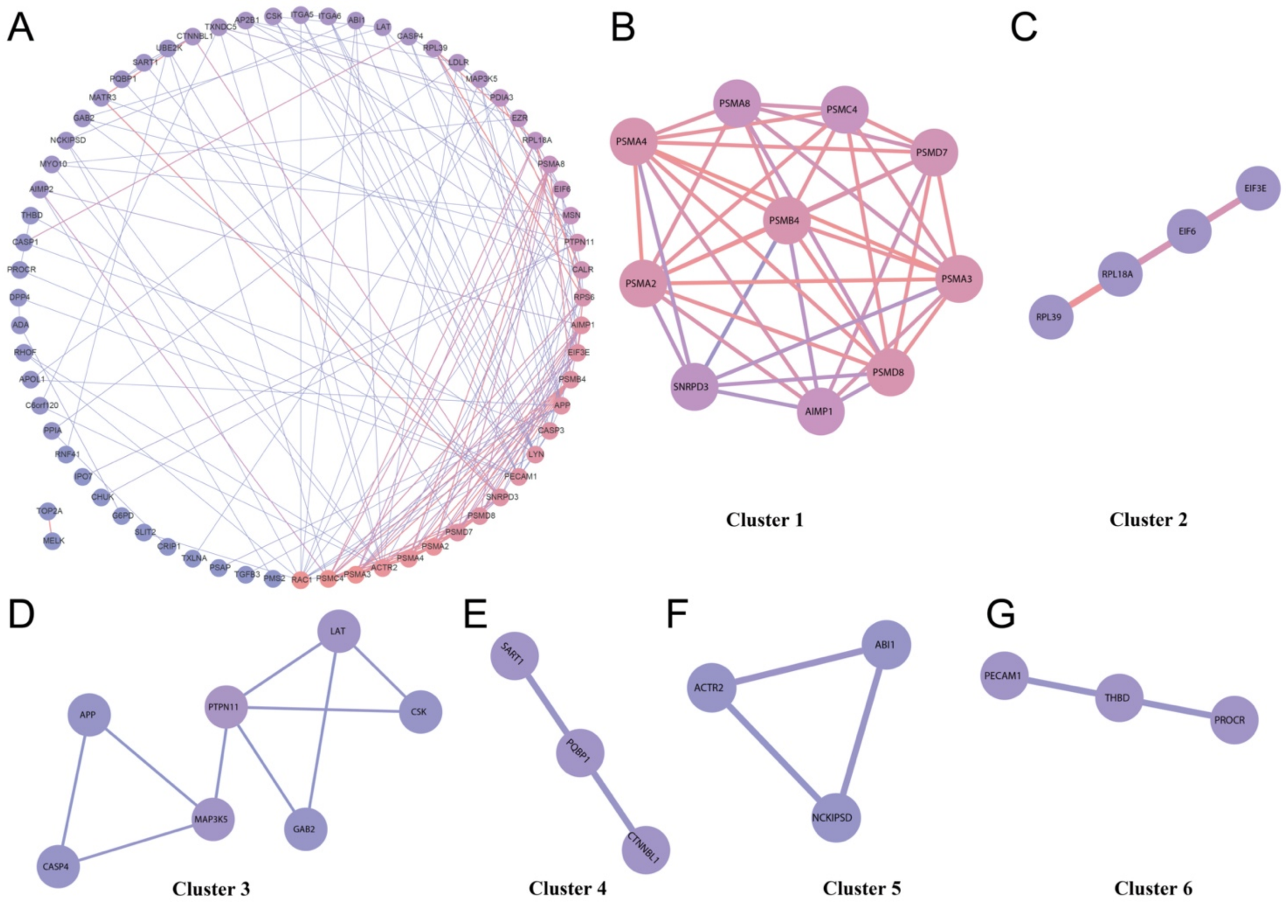

Supplementary Figure 2. PPI network reveals several immune-related modules. (A) PPI network of differentially expressed immune-associated genes. **(B-G)** Six modules may play an important role in the development of OSCC. The redder the edge, the higher the co-expression, and the redder the dot, the higher the degree. ppi refers to protein interactions.

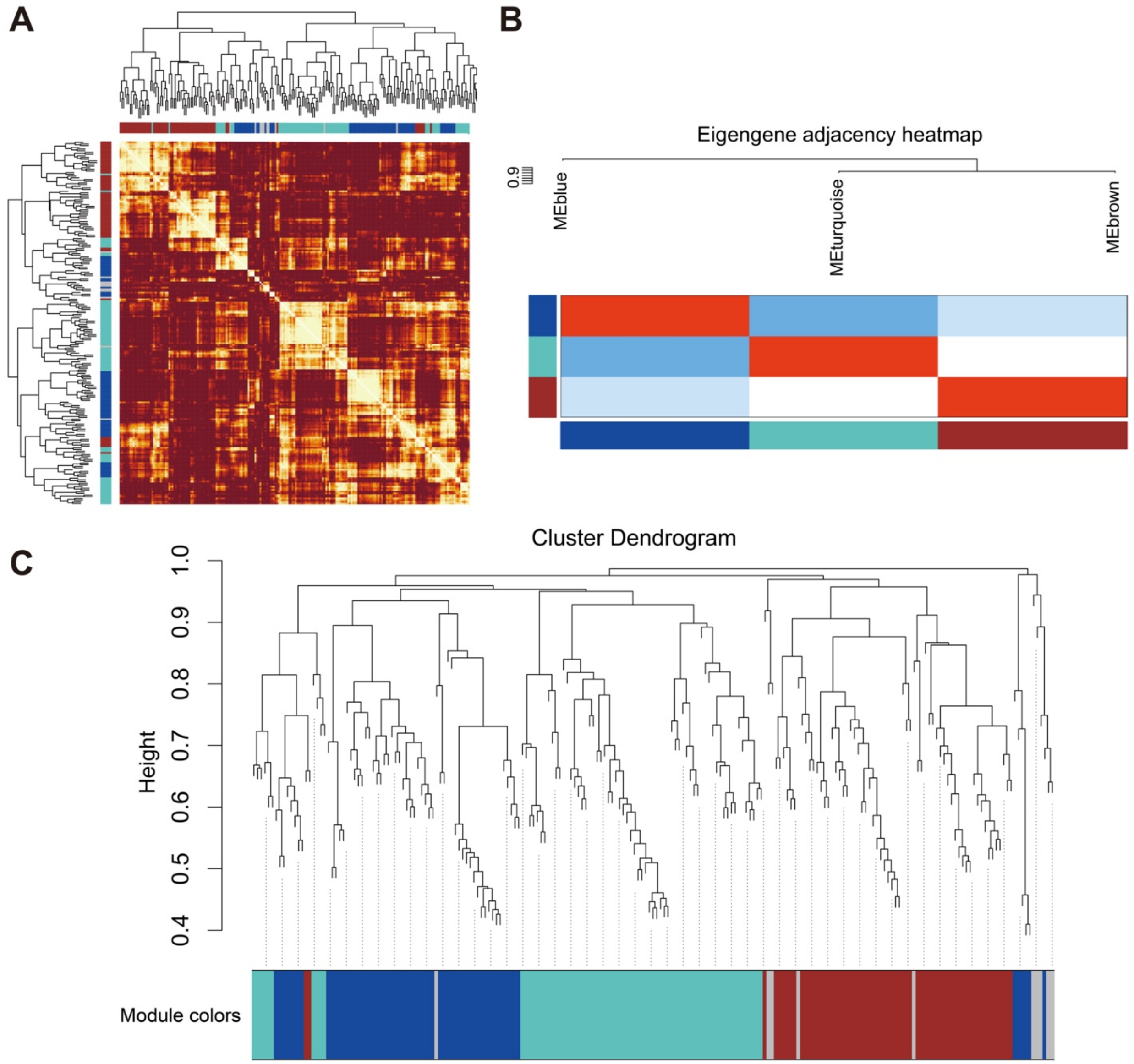

Supplementary Figure 3. Key module correlated with OSCC identified by WGCNA. (A) The heatmap depicts the TOM among all genes in the analysis. **(B)** Clustering of all modules. **(C)** Cluster dendrogram of genes.

2 Supplementary Tables

Supplementary Table 1. Primer sequences for RT-qPCR

Gene	Forward primer (**5′-3′**)	Reverse primer (**5′-3′**)
α-SMA	AGCGTGGCTATTCCTTCGTT	GCCCATCAGGCAACTCGTAA
FAP	TTATGCTGGTCGCCTGTTGG	AGGAGACCACCAGAGAGCATA
β-tublin	TCTCCTTATCAGCAAGATCCGAG	TTCAGATCCCCGTAGGTTGGT
PIGR	TTGTCATCAACCAACTCAGGCTCAG	AGCACTTGGAGGTCAGCATTCTTC
CD81	TCGCCAAGGATGTGAAGCAGTTC	GCAGTCAAGCGTCTCGTGGAAG
PTTG1IP	AACCTGTGAAGAGTGCCTGAAGAAC	AGGAAGCGTCGGGACTGATGTG
UACA	CCATTGGAACAGGTTGAGGCTCTG	GGTCACTGTCTGCTGCTCTTTCTC
ACTR2	CACAGAACCTCCTATGAACCCAACC	ACAAAGTCAGAACTGCCTGGATGG
EIF6	GGATGGTGGTGAATGACTGGTGTG	CAATGGTGCTAGGCTGGGCTTC
GAPDH	GAAGGTCGGAGTCAACGGATTT	GCCATGGGTGGAATCATATTGG
