# Supplementary figures and images for: Analysis of mRNA-miRNA interaction network reveals the role of CAFs-derived exosomes in the immune regulation of oral squamous cell carcinoma
Source: BMC Cancer. 2023 Jun 26;23:591. doi: 10.1186/s12885-023-11028-5 (PMC10294399; doi:10.1186/s12885-023-11028-5)

Figure 1C

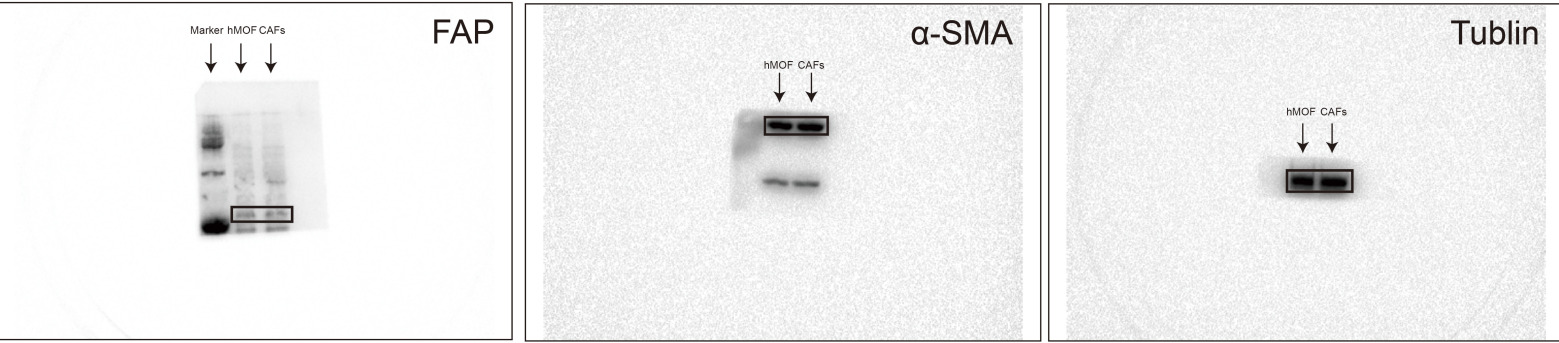

Figure 1E

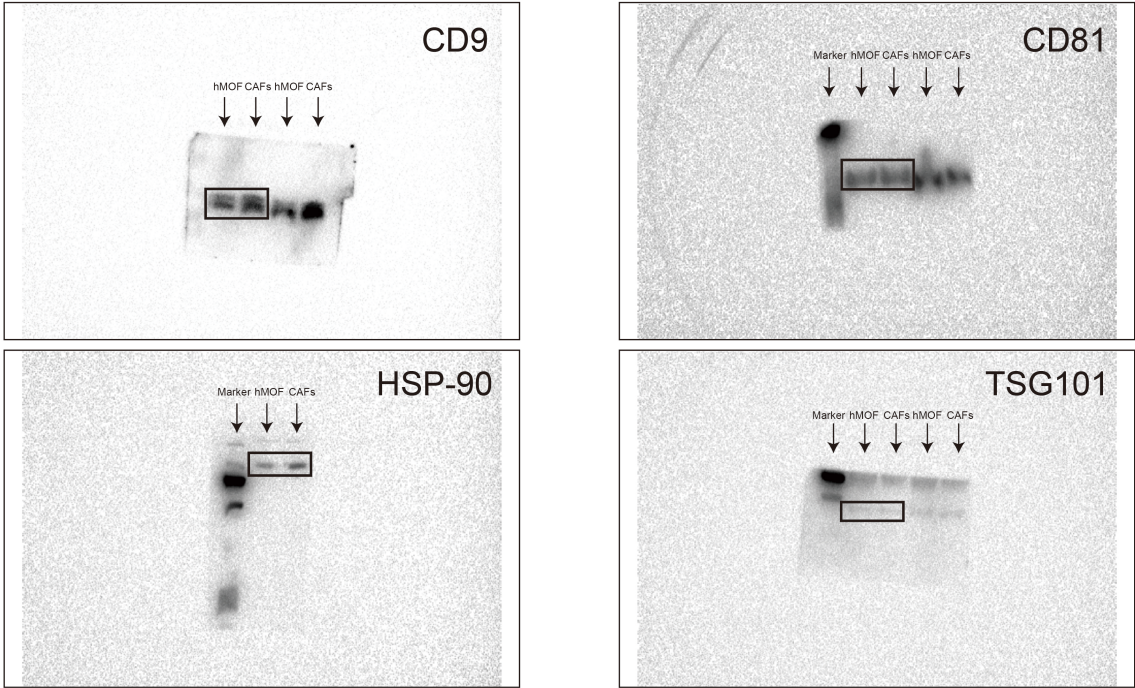

Figure 2E

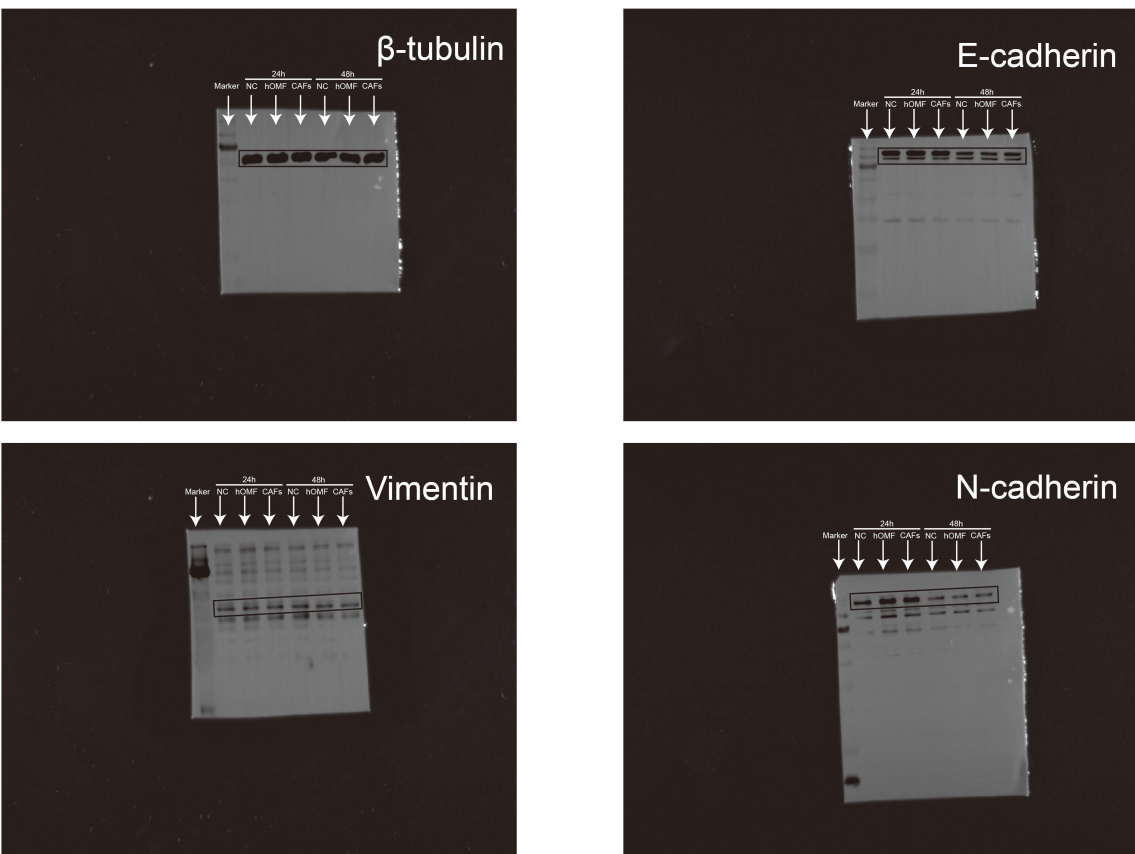

Supplement: Supplementary file 2 — Additional file 2. [file 12885_2023_11028_MOESM2_ESM.pdf]
